# Peripheral blood tRNA-derived fragments as novel noninvasive biomarkers for diagnosis and prognostic stratification in multiple myeloma

**DOI:** 10.3389/fimmu.2025.1650510

**Published:** 2025-12-03

**Authors:** Biyun Yi, Yan Gao, Yanzhao Huang, Kaiyun Guo, Rong Liu, Mengying Zeng, Han Xu, Ming Lei

**Affiliations:** 1 Changde Hospital, Xiangya School of Medicine, Central South University (The First People’s Hospital of Changde City), Changde, China; 2 Hengyang Medical College, University of South China, Hengyang, China

**Keywords:** tsRNA, multiple myeloma, peripheral blood mononuclear cell, diagnostic biomarkers, tsRNA-Notch signaling pathway target

## Abstract

**Background:**

Transfer RNA-derived small RNAs (tsRNAs) have recently emerged as critical regulators in cancer biology; yet their expression profiles and clinical relevance in multiple myeloma (MM) remain poorly defined.

**Methods:**

High-throughput sequencing was performed to comprehensively characterize tsRNA expression profiles in peripheral blood mononuclear cells (PBMCs) from 22 newly diagnosed MM patients and 19 healthy controls. Shared target genes were predicted by integrating miRanda and TargetScan, and a tsRNA–mRNA interaction network was constructed; GO and KEGG functional enrichment analyses were also performed. The discriminative performance of candidate biomarkers was evaluated using receiver operating characteristic (ROC) curves, and the associations between candidate tsRNAs and clinical parameters were further examined by correlation analyses.

**Results:**

A total of 148 significantly upregulated and 63 downregulated tsRNAs were identified. Among them, Other-1_19-tRNA-SeC-TCA-1 and Other-36_54-tRNA-Met-CAT-2-M4 exhibited excellent diagnostic performance, with areas under the ROC curve (AUCs) of 0.9557 and 0.9773, respectively. Functional analyses revealed that differentially expressed tsRNAs were primarily involved in regulating signaling pathways such as TGF-β, PI3K-Akt, and AMPK, and were closely associated with thyroid hormone metabolism. Elevated expression of Other-22_52-tRNA-Gly-GCC-1-M3 was significantly correlated with renal insufficiency (p < 0.05), suggesting its potential as a novel biomarker for assessing renal injury risk. The tsRNA–mRNA regulatory network comprised 11,141 interaction edges and exhibited a power-law topology, with the hub gene SRSF10 mediating coordinated regulation across multiple pathways.

**Conclusions:**

Our findings reveal a distinct imbalance in tsRNA expression in MM, indicating that specific tsRNA fragments contribute to disease progression and renal injury through multifaceted signaling networks, thereby providing novel molecular insights for early diagnosis and risk stratification.

## Introduction

Multiple myeloma (MM) is a malignant hematological tumor characterized by abnormal clonal proliferation of plasma cells (PCs), primarily affecting the bone marrow and skeletal system (1). Its clinical manifestations include bone pain, anemia, hypercalcemia and renal impairment, seriously impacting patients’ quality of life and survival (2). Although novel therapeutic agents such as proteasome inhibitors, immunomodulators and monoclonal antibodies have significantly improved the prognosis of MM patients in recent years, the disease remains incurable and prone to drug resistance and recurrence (3–5). Therefore, in-depth exploration of MM pathogenesis to identify novel diagnostic and therapeutic targets, are of substantial clinical significance.

Traditional diagnostic approaches for multiple myeloma (MM), including serum M-protein quantification and bone marrow examination, often suffer from limited sensitivity, particularly in detecting early lesions or minimal residual disease. Similar challenges have been encountered in other malignancies. For instance, in thyroid cancer, the concept of disease-specific molecular targeting has been successfully implemented through novel radiopharmaceuticals (e.g., PSMA, SSTR, and FAP), which have markedly enhanced diagnostic sensitivity and therapeutic precision when conventional imaging modalities reach their functional limits. This paradigm suggests that identifying molecular biomarkers with disease-specific biological relevance may help overcome the limitations of traditional diagnostic techniques (6). Building on this rationale, we aimed to explore disease-specific noncoding RNAs, particularly tRNA-derived fragments (tRFs), as novel, noninvasive biomarkers for the diagnosis and prognostic stratification of MM.

Abnormal regulation of gene expression plays a critical role in tumorigenesis and progression (7–10). As important molecules in gene expression regulation, non-coding RNAs (ncRNAs) have attracted much attention in recent years (11–13). Transfer RNA (tRNA)-derived small RNAs (tsRNAs), a recently identified class of ncRNAs (18–40 nucleotides in length), are generated from tRNAs through specific enzymatic cleavage (14, 15). Based on their origin and splicing sites, tsRNAs are classified into tRNA⁃derived fragments (tRFs) and tRNA halves (tiRNAs) (16). Studies have revealed that tsRNAs exert profound impacts on tumor cell behavior through mechanisms such as regulating gene expression, participating in epigenetic modifications, and modulating signaling pathways (17, 18). They directly interact with transcription factors and chromatin-modifying enzymes to alter chromatin states and transcriptional activity, while also engage with other ncRNAs to form extensive gene regulatory networks (19). The tumor-specific expression patterns and functional roles of tsRNAs provide promising avenues for early diagnosis, therapeutic effect evaluation, and prognosis assessment of tumors.

It has been demonstrated that tsRNAs play crucial roles in a variety of cancers. Dhahbi et al. identified breast cancer-associated tsRNAs, particularly 5′ tiRNAs and YRNA fragments, through deep sequencing of serum samples. They revealed distinct tsRNA expression patterns that significantly differed from healthy controls (HCs), with isoform levels potentially correlating with disease progression (20). Similarly, Zhu et al. reported significantly elevated tsRNA levels in plasma exosomes of liver cancer patients were higher than those of HCs, and identified four enriched tsRNAs, including tRNA-ValTAC-3, tRNA-GlyTCC-5, tRNA-ValAAC-5, and tRNA-GluCTC-5 (21). These studies suggested the potential of tsRNAs as novel biomarkers and therapeutic targets for the diagnosis and treatment of cancer. However, the expression profiles, function and related mechanisms of tsRNAs in MM remain to be investigated.

Renal impairment is a common and severe complication of MM, substantially contributing to increased morbidity and mortality. However, the molecular mechanisms underlying MM-associated renal injury remain poorly understood. Given the emerging regulatory roles of tsRNAs in tumor biology and cellular stress responses, their dysregulated expression may contribute to renal dysfunction in MM, providing new insights into the molecular pathways that connect MM progression with renal damage.

In this study, high-throughput sequencing of tsRNAs was performed on peripheral blood mononuclear cells (PBMCs) obtained from patients with MM and healthy controls (HCs) to identify differentially expressed tsRNAs. Selected candidates were subsequently validated by quantitative RT-PCR, and their clinical relevance was analyzed to assess potential value in MM diagnosis and prognosis. In addition, the association between tsRNA expression levels and the risk of MM-related renal impairment was examined to explore their potential utility in the early detection of complications. Finally, bioinformatic analyses were conducted to predict potential target genes and construct regulatory networks, providing molecular insights into the mechanisms through which tsRNAs may contribute to MM pathogenesis.

## Materials and methods

### Clinical samples

We enrolled 22 patients with newly diagnosed multiple myeloma and 19 healthy controls, all of whom were confirmed through comprehensive medical examinations. Peripheral blood mononuclear cells (PBMCs) were subsequently collected from all participants for analysis. Three samples per group were randomly selected for small RNA sequencing on the Illumina platform; the remaining specimens were reserved for RT-qPCR validation. Inclusion criteria were listed as follows: (1) Newly diagnosed, treatment-naive MM patients meeting the diagnostic criteria of the Chinese Guidelines for the Diagnosis and Management of Multiple Myeloma (2020 revision); (2) Agree to sign the informed consent forms. Exclusion criteria comprised patients with severe infection, chronic liver disease, renal insufficiency, autoimmune diseases organ failure, other malignancies, or hematologic disorders. This study was approved by the Ethics Committee of Changde First People’s Hospital.

### Total RNA extraction by TRIzol

Total RNA was extracted from PBMCs using TRIzol (Invitrogen, CA, USA). The integrity of each RNA sample was checked by agarose gel electrophoresis, and its quantity and concentration were examined using Nanodrop ND-1000 instrument (Nano Drop Thermo, DE, USA).

### Library preparation & sequencing

RNA modifications of the fragments derived from tRNA (tRF & tiRNA) were removed to avoid interfering library preparation of small RNA-sequencing. The processes of library preparation were following: the ligation to adapters of small RNA, cDNA synthesis and amplification, the purification of 134∼160 bp fragments, and finally the library quantification using Agilent 2100 Bioanalyzer. After the denaturation and dilution of libraries, tRF & tiRNA were sequenced by Illumina sequencer.

### Sequencing data analysis

The quality of raw sequencing data was tested by FastQC (version 0.11.7)(http://www.bioinformatics.babraham.ac.uk/projects/fastqc/.2010). Trimmed reads of 5’, 3’-adaptor bases were obtained using Cutadapt (version 1.17) (22), and only one mismatch was allowed when aligned to mature tRNA sequences. Only one mismatch of reads that don’t map was allowed when aligned to precursor tRNA sequences with Bowtie software (version 1.2.2) (23), and the remaining was aligned to miRNA sequences with miRDeep2 (version 2.0.0.8) (24). According to the statistical analysis of alignment, the results were selected for sequencing data analysis. The expression profiles and differential expression of tRFs & tiRNAs and miRNAs were acquired and analyzed using the package of R (version 3.5.1) (25) and Perl (version 5.16.3), including Correlation Analysis, Principal Component Analysis (PCA), Pie plots, Venn plots, Volcano plots, Scatter plots, and Hierarchical clustering.

### RT-qPCR validation

After RNA extraction, its quality and quantity were assessed using agarose gel electrophoresis and the Nanodrop ND-1000 instrument. The extracted RNA was pretreated with the rtStar™ tRF & tiRNA Pretreatment Kit (Cat# AS-FS-005, Arraystar) and reverse transcribed into cDNA using the rtStar™ First-Strand cDNA Synthesis Kit (3’ and 5’ adaptors) (Cat# AS-FS-003, Arraystar). Real-time quantitative PCR (RT-qPCR) was performed on the QuantStudio™ 5 Real-Time PCR System (Applied Biosystems). The expression levels of target genes were normalized to that of the reference gene U6. The primer sequences for U6, Other-1_19-tRNA-SeC-TCA-1(tRF-SeC-TCA-1),Other-22_52-tRNA-Gly-GCC-1-M3(tRF-Gly-GCC-1-M3),and Other-36_54-tRNA-Met-CAT-2-M4 (tRF-Met-CAT-2-M4) are provided in **Supplementary Table S1**.

### Bioinformatic analysis

The target gene predictions and functional enrichment analysis of Kyoto Encyclopedia of Genes and Genomes (KEGG) Pathway and Gene Ontology (GO) Biological Processes, were carried out to indicate the biological function of the differentially expressed tRFs & tiRNA, mainly focusing on three tRNAs tRF-SeC-TCA-1,tRF-Gly-GCC-1-M3, tRF-Met-CAT-2-M4. TargetScan and Miranda algorithms were applied for target gene predictions. The KEGG database (http://www.kegg.jp/) was used for the pathway enrichment analysis.

### Statistical analysis

SPSS (version 18.0) was used to conduct statistical analysis. The Mann-Whitney U test was used to analyze the differential expression of genes between the experimental and control groups. Pearson correlation coefficient (*r*) was used to verify the correlations between the three genes and other clinical indicators. Additionally, receiver operating characteristic (ROC) curves and correlation heatmaps were plotted using Prism.

## Results

### Differential expression of tRFs & tiRNAs in PBMCs of MM patients

**Figure 1:**
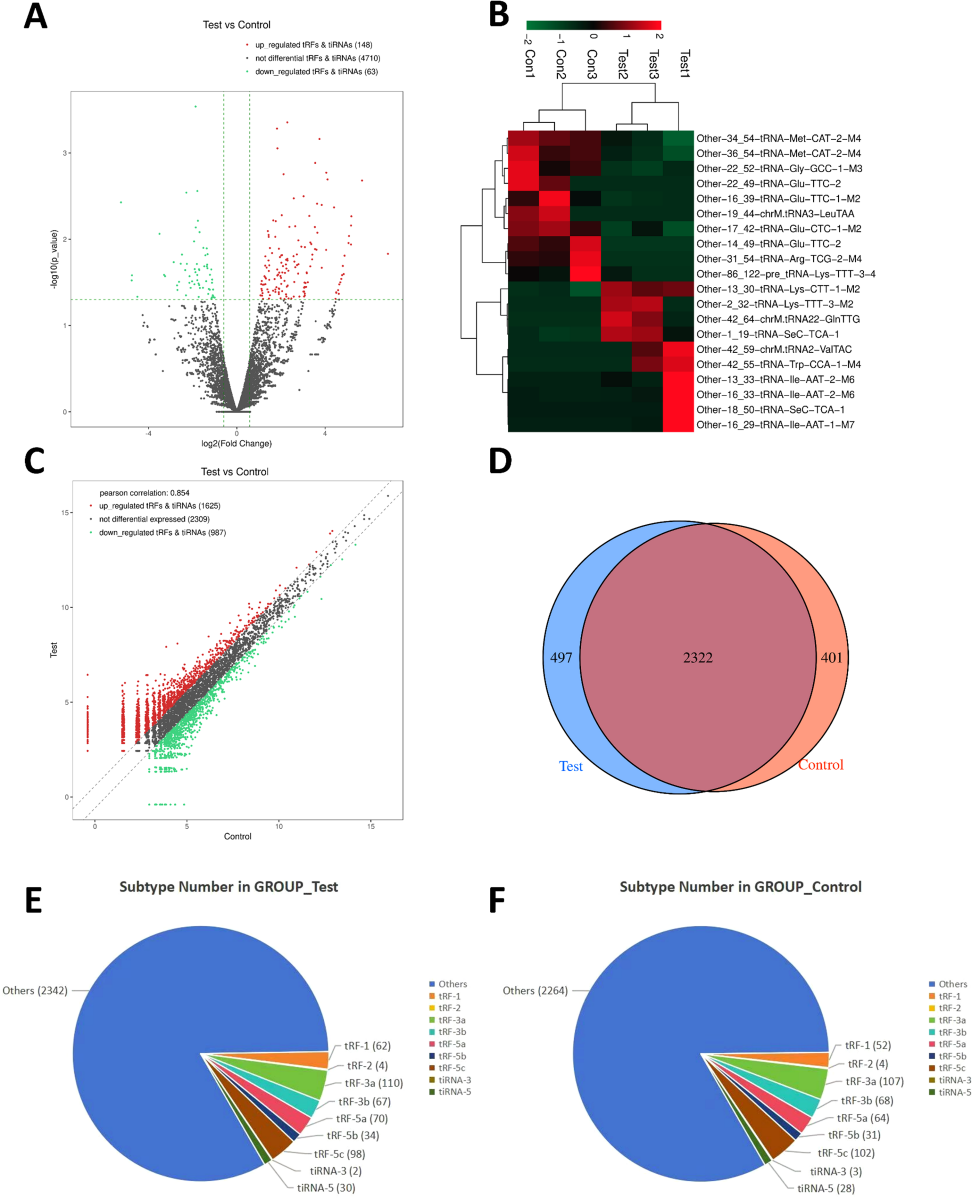
Analysis of differentially expressed tsRNAs in PBMCs from MM patients and HCs. **(A)** Volcano plot analysis of differentially expressed tsRNAs between MM patients (Test) and controls (Control). Upregulated tRFs & tiRNAs (n = 148; positive X-axis) and downregulated tRFs & tiRNAs (n = 63; negative X-axis) show similarly high Y-values. Gray dots (n = 4710) represent non-significant expressed tRFs & tiRNAs. **(B)** Hierarchical clustering heatmap of small RNA expression profiles. Expression changes of tRFs & tiRNAs are color-coded: red (upregulated), green (downregulated), black (unchanged). Color saturation represents the degree of expression change. **(C)** Scatter plot of tRF & tiRNA expression. Red (n = 1625), green (n = 987), and black (n = 2309) dots represent upregulated, downregulated, and unchanged tsRNAs, respectively. **(D)** Venn diagram of expressed tRFs & tiRNAs. Intersection shows 2322 shared tsRNAs; left blue circle contains 497 MM-specific tsRNAs; right red circle shows 401 tsRNAs control-specific tsRNAs. **(E, F)** Pie charts of tsRNA subtype distribution in **(E)** MM patients and **(F)** controls. “Other” represents unclassified tsRNA fragments.

High-throughput sequencing of tRFs & tiRNAs from three pairs of MM patients and matched HCs identified a total of 4,921 unique sequences. Among these, 4,815 were novel, 106 matched the known entries in the tRF database (tRFdb), and 46 previously catalogued tRFdb sequences were not detected (**Supplementary Figure S1A**). Based on this, we conducted a detailed analysis to quantified the differential expression levels of these sequences by calculating the log_2_ fold change (log_2_FC) values for significantly differentially expressed RNAs. The results showed that substantial differences in small RNA expression profiles between MM patients and controls, identifying 148 significantly upregulated and 63 significantly downregulated tRFs & tiRNAs in the MM group. These differentially expressed tsRNAs were visualized through volcano plot and hierarchical clustering heatmap analyses (**Figures 1A, B**). An unsupervised hierarchical clustering heatmap of row Z-score-normalized expression data clearly distinguished MM samples from HCs, forming two distinct clusters. This analysis revealed substantial molecular heterogeneity within key gene networks between the two groups (**Supplementary Figure S1B**). The Sample Correlation Heatmap demonstrated strong correlations of tRF & tiRNA expression levels across samples, with Pearson *r* approaching 1, indicating highly consistent expression patterns (**Supplementary Figure S1C**). PCA analysis revealed clear separation between MM and control groups across the first three principal components (PC1-3), highlighting a clear distinction in gene expression profiles (**Supplementary Figure S1D**). The scatter plot showed a strong correlation (Pearson *r* = 0.854) in overall expression levels between groups, despite significant differential expression of individual tRFs & tiRNAs (**Figure 1C**).

Venn diagram showed distinct tRF & tiRNA profiles between MM patients and controls (**Figure 1D**). Notably, 2322 tsRNAs were co-expressed in both groups, potentially participating in fundamental cellular processes. The 497 MM-specific and 401 control-specific tsRNAs might contribute to MM pathogenesis or normal physiological functions, respectively. Subtype distribution analysis demonstrated tRF-3a and tRF-5c were predominantly expressed in both groups (**Figures 1E, F**), indicating stable expression across biological conditions. Unclassified tsRNAs (“Other” category) constituted the largest proportion (MM: 2342; controls: 2264), reflecting the diversity and complexity of tsRNAs. Despite quantitative differences in unclassified tsRNAs between groups, most tsRNAs exhibited minimal expression variation across conditions.

### Subtype and length distribution of tRFs & tiRNAs profiles in MM patients

**Figure 2:**
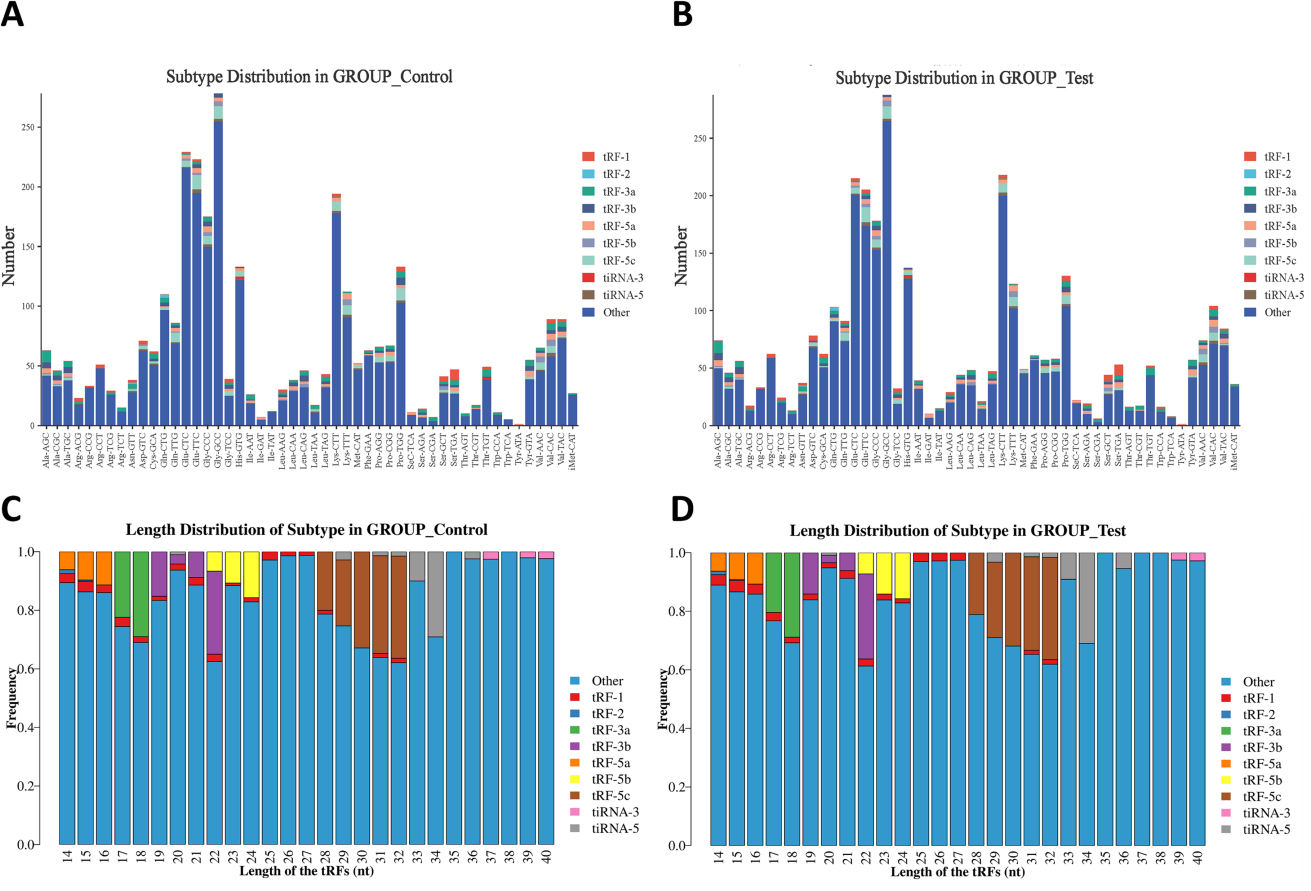
Differences and characterizations of subtype and length distribution of tRFs & tiRNAs between MM patients and controls. **(A, B)** Subtype distribution of tRFs & tiRNAs in **(A)** controls (GROUP_Control) and **(B)** MM patients (GROUP_Test). **(C, D)** Length distribution of tRFs & tiRNAs in **(C)** controls and **(D)** MM patients.

After Solexa CHASTITY filtering and 3’ adapter removal, the unique read counts of tsRNA subtypes in the GROUP_Control and GROUP_Test samples were analyzed. Stacked bar charts revealed that the “Other” category constituted the primary component in both groups, showing highly consistent distribution patterns. In GROUP_Control, prominent peaks occurred at tRNA sites including Gly-GCC, Glu-CTC, and Glu-TTC, a pattern similarly observed in GROUP_Test. Notably, tRF-5a, tRF-5b, and tRF-5c subtypes showed marked upregulation in GROUP_Test, suggesting differential expression between groups (**Figures 2A, B**).

Length distribution analysis of tRFs & tiRNAs revealed that the “Other” category was predominant across all length intervals (14–40 nucleotides (nt)), particularly abundant at 18–22 nt and 30–32 nt (**Figures 2C, D**). Specific subtypes (tRF-1, tRF-2, tRF-3a, tRF-3b, tRF-5a, tRF-5b, tRF-5c, tiRNA-3, and tiRNA-5) exhibited distinct length dependencies. Notably, tRF-3b, tRF-5c, and tiRNA-3 showed significant enrichment at 18–22 nt and 30–34 nt, suggesting potential functional specialization. While both groups shared similar overall distribution trends, GROUP_Test displayed significantly higher tRF-5c abundance at 30~32 nt compared to GROUP_Control, suggesting that MM-associated expression alterations might reflect its potential regulatory role in disease pathogenesis.

### Target gene prediction and relationship network construction of MM-associated tsRNAs

Based on stringent screening criteria (log_2_FC > 2, p < 0.05, CPM > 50), eight tsRNAs were identified as significantly differentially expressed in multiple myeloma (MM), including Other-16_33-tRNA-iMet-CAT-1-M2, Other-13_30-tRNA-Lys-CTT-1-M2, Other-1_19-tRNA-SeC-TCA-1, Other-22_52-tRNA-Gly-GCC-1-M3, Other-36_54-tRNA-Met-CAT-2-M4, Other-16_39-tRNA-Glu-TTC-1-M2, Other-34_54-tRNA-Met-CAT-2-M4, and Other-17_42-tRNA-Glu-CTC-1-M2 (**Supplementary Table S2**).

**Figure 3:**
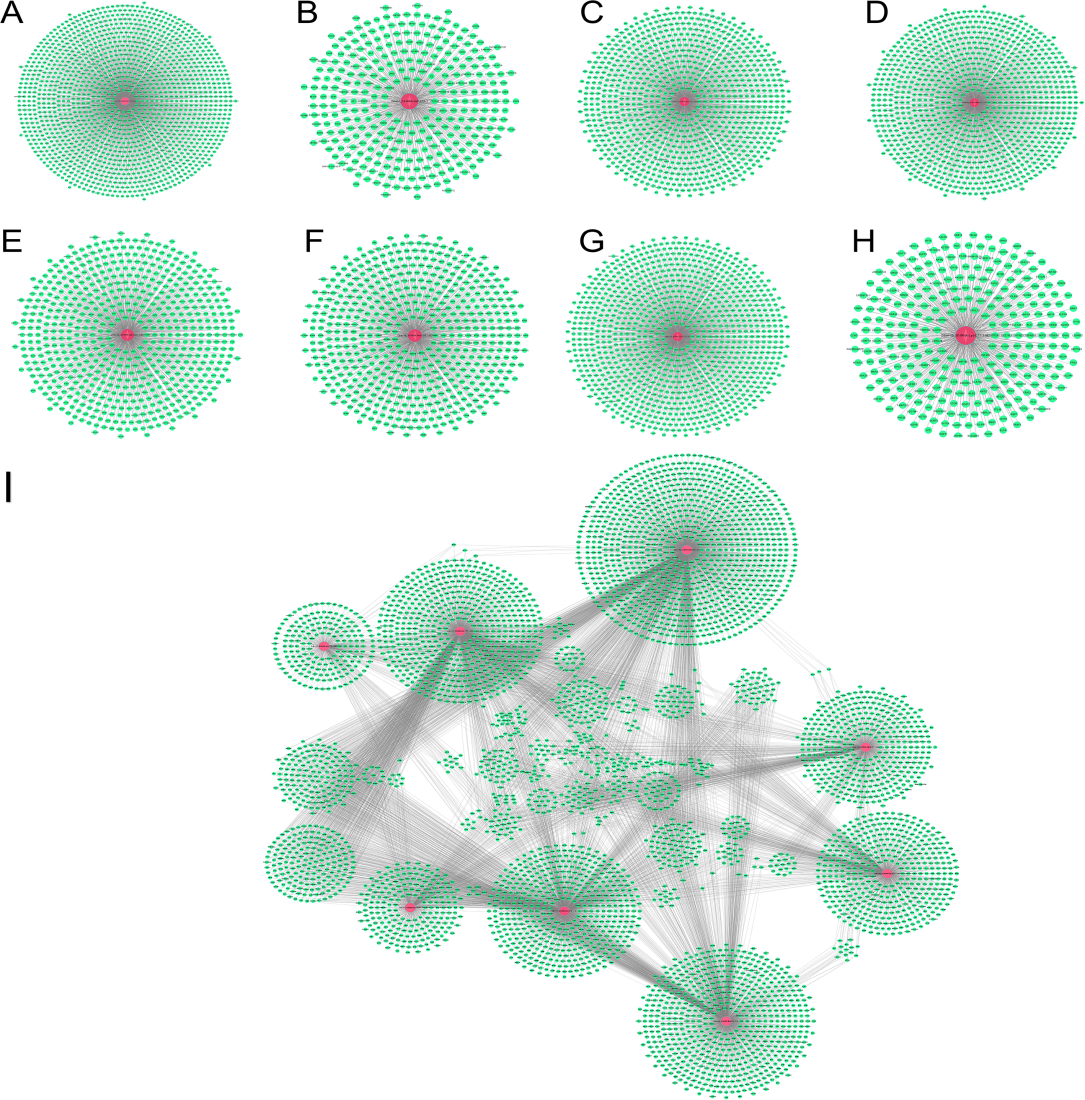
Target gene prediction of MM-associated tsRNAs and tsRNA-target gene regulatory network construction. **(A–H)** The predicted target genes of eight significantly differentially expressed tsRNAs, including Other-36_54-tRNA-Met-CAT-2-M4,Other-1_19-tRNA-SeC-TCA-1, Other-17_42-tRNA-Glu-CTC-1-M2,Other-34_54-tRNA-Met-CAT-2-M4, Other-22_52-tRNA-Gly-GCC-1-M3,Other-16_33-tRNA-iMet-CAT-1-M2, Other-16_39-tRNA-Glu-TTC-1-M2 and Other-13_30-tRNA-Lys-CTT-1-M2 respectively. **(I)** The tsRNA-target gene regulatory relationship network of candidate tsRNAs. Red nodes represent the tsRNAs, green nodes represent target genes, and the connecting lines indicate interactions between tsRNAs and target genes.

Integration of the miRanda and TargetScan algorithms predicted 1,112 potential target genes for the three upregulated tsRNAs (**Figures 3B, F, H**), and 4,190 potential targets for the five downregulated tsRNAs (**Figures 3A, C–E, G**). Each tsRNA exhibited a radial topology centered on a core node, with approximately 400–1,000 predicted targets per tsRNA. Among them, Other-1_19-tRNA-SeC-TCA-1 and Other-13_30-tRNA-Lys-CTT-1-M2 displayed the highest degrees of connectivity, suggesting their potential roles as key post-transcriptional regulators across multiple signaling pathways.

After removing redundant targets, a total of 5,009 unique genes were retained to construct the tsRNA–mRNA regulatory network (**Figure 3I**), comprising 8 tsRNA nodes, 5,009 gene nodes, and 11,141 regulatory edges. This network exhibited scale-free and modular topological characteristics typical of biological regulatory systems. Several high-degree hub genes, such as SRSF10, MPZL1, OSBP, SERF2, ARHGAP5, and SLC35A3, formed localized star-like subnetworks. Cross-links among tsRNAs through shared target genes generated functionally coherent modules, suggesting potential synergistic or coupled regulatory mechanisms among tsRNAs in MM.

Collectively, these findings demonstrate that tsRNA-mediated regulation in MM exhibits a hierarchical and functionally selective organization. The central hubs are enriched for genes involved in core signaling and RNA processing pathways, whereas the peripheral nodes are associated with specific biological processes. This hierarchical organization provides new insights into the mechanistic roles of tsRNAs in the molecular pathogenesis of multiple myeloma.

### Functional enrichment analysis of tsRNA target genes

**Figure 4:**
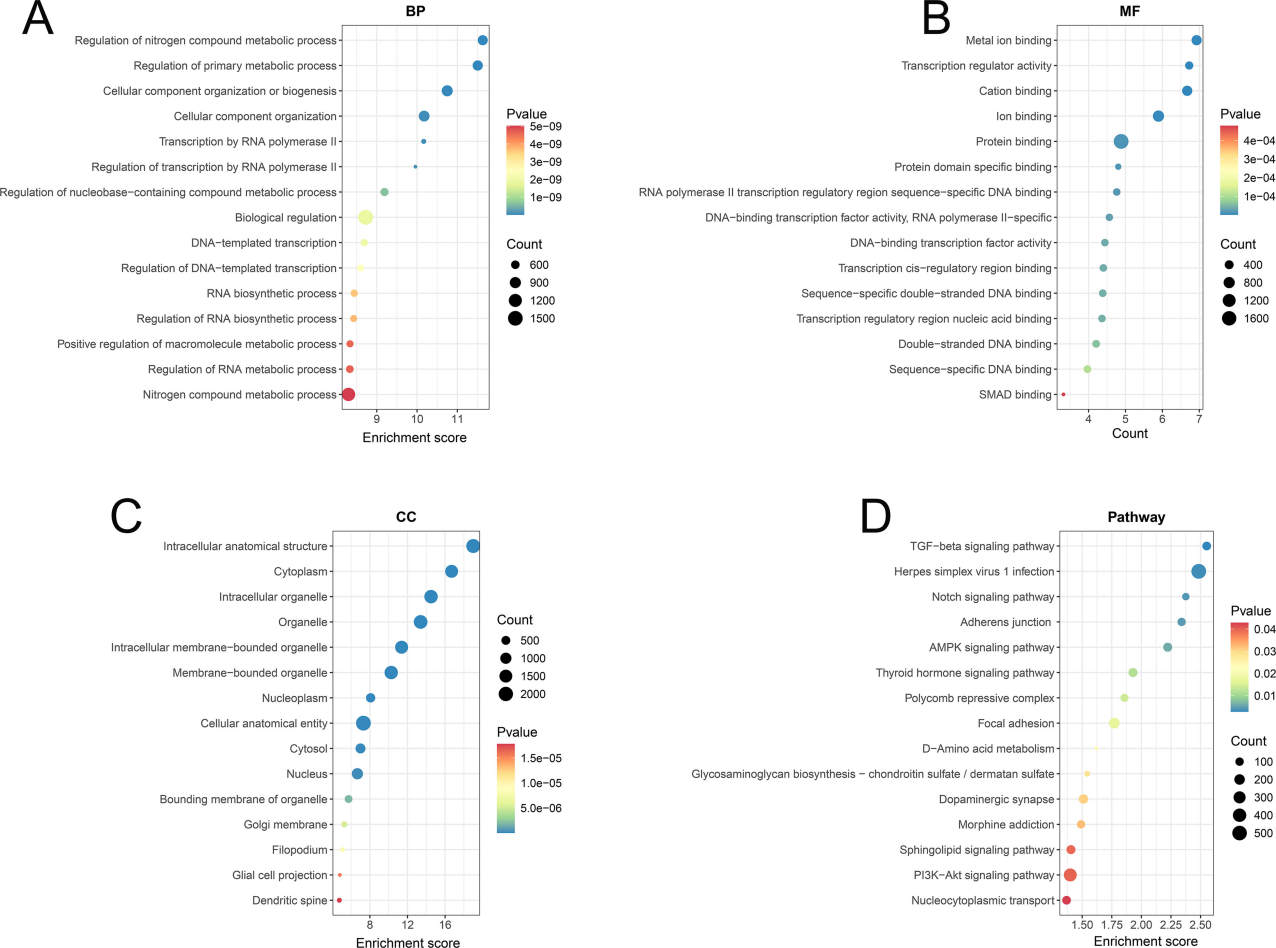
Functional enrichment analysis of tsRNA target genes in MM patients from GO and KEGG datasets. **(A–C)** The GO enrichment analysis of tsRNA target genes in MM patients across three categories-biological process (BP), molecular function (MF), and cellular component (CC). **(D)** The KEGG pathway enrichment analysis of tsRNA target genes in MM patients.

To further elucidate the functions of the differentially expressed tsRNA target genes, we performed GO enrichment analyses on all predicted target genes. The GO enrichment analysis was conducted across three categories-biological processes (BPs), molecular functions (MFs), and cellular components (CCs) (**Figures 4A–C**). The BPs demonstrated enrichment for “regulation of nitrogen compound metabolic process” (GO:0051171), “regulation of primary metabolic process” (GO:0080090), “cellular component organization or biogenesis” (GO:0071840), “cellular component organization” (GO:0016043), “transcription by RNA polymerase II” (GO:0006351) and “regulation of transcription by RNA polymerase II” (GO:0006366), indicating the target genes were primarily involved in critical processes such as nitrogen compound metabolism, RNA polymerase II transcription, and ribosome biogenesis, which reflects their central role in the regulation of metabolism and gene expression.

Besides, the MFs were primarily associated with “metal ion binding” (GO:0046872), “transcription regulator activity” (GO:0140110), “cation binding” (GO:0043169), “ion binding” (GO:0043167), “protein binding” (GO:0005515) and “protein domain-specific binding” (GO:0019904), indicating that the target genes were enriched in functions such as metal ion binding, DNA binding, protein binding, and transcriptional regulation, highlighting their regulatory roles in gene transcription and protein interactions. The CCs showed localization to “intracellular anatomical structure” (GO:0005622), “cytoplasm” (GO:0005737), “intracellular organelle” (GO:0043229), “organelle” (GO:0043226), “intracellular membrane-bounded organelle” (GO:0043231) and “membrane-bounded organelle” (GO:0043227), suggesting that the target genes were mainly localized in the cytoplasm, organelles, nucleus, and nuclear membrane.

The results of the KEGG pathway enrichment analysis were shown in **Figure 4D** and **Supplementary Figure S2**. The target genes of differential tsRNAs were predominantly enriched in the TGF-beta signaling pathway (hsa04350), Notch signaling pathway (hsa04330), Adherent junction (hsa04520), AMPK signaling pathway (hsa04152), Thyroid hormone signaling pathway (hsa04919) and PI3K-Akt signaling pathway (hsa04151), revealing they were predominantly enriched in several crucial signaling pathways involving in cell proliferation, differentiation and immune responses, such as the TGF-beta, Notch, and PI3K-Akt signaling pathways. Further analysis revealed that distinct tsRNAs exhibit specific pathway enrichment profiles. The target genes of Other-1_19 is predominantly associated with parathyroid hormone synthesis, secretion and action, dilated cardiomyopathy and circadian entrainment. The target genes of Other-22_52 shows significant enrichment in herpes simplex virus 1 infection pathway. The target genes of Other-36_54 is significantly enriched in sphingolipid signaling, TGF-β signaling, dopaminergic synapses, AMPK signaling and adherens junction. Collectively, these observations supported the functions of differentially expressed tsRNAs in the pathogenesis and progression of MM through the coordinated modulation of cellular metabolism, gene expression, and intracellular signaling networks.

### Functional enrichment analysis of differentially expressed tsRNAs and their potential biological roles

**Figure 5:**
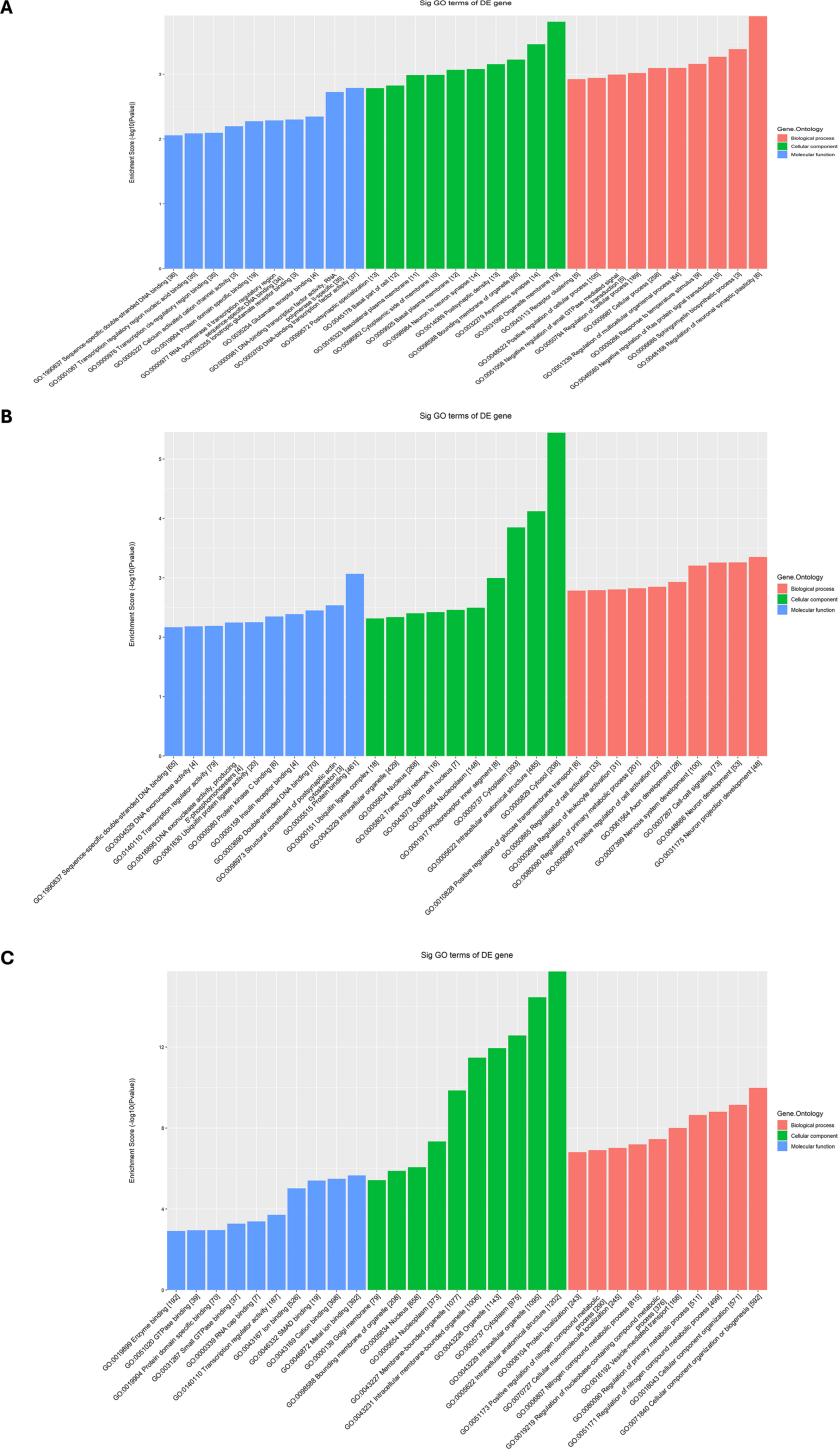
Functional enrichment analysis of three differential expressed tsRNAs in MM patients from GO datasets, including **(A)** tRF-SeC-TCA-1, **(B)** tRF-Gly-GCC-1-M3 and **(C)** tRF-Met-CAT-2-M4.

To investigate the potential roles of differentially expressed tsRNAs in MF, CC, and BP, three tsRNAs were selected for GO enrichment analysis (**Figures 5A–C**). According to the selection criteria, tsRNAs with higher FC and smaller *p*-values were prioritized to ensure significant differences. tsRNAs longer than 20 nt were chosen to facilitate PCR primer design. tsRNAs with consistent CPM values were selected to minimize experimental interference from low expression levels. Additionally, tsRNAs derived from the cytoplasm were prioritized due to their critical roles in cell proliferation and metabolism. The selected tsRNAs included tRF-SeC-TCA-1,tRF-Gly-GCC-1-M3, tRF-Met-CAT-2-M4. (**Figures 5A–C**). The GO enrichment analysis indicated that the Other-1 group is localized in the nucleus, and play roles in DNA and RNA binding, DNA replication and chromosome structure maintenance. Its BPs are enriched in the regulation of gene expression and coordination of transcription and replication, highlighting their critical functions in nuclear genome stability and transcriptional regulation. The Other-22 group is localized in the cytoplasm and ribosomes, and mainly associated with RNA binding and transcription factor activity. Its MFs are enriched in protein translation and nuclear-cytoplasmic regulatory pathways, and the BPs are involved in cell cycle regulation and transcriptional activation, indicating their central roles in maintaining cell proliferation and metabolic homeostasis. Besides, the Other-36 group that is localized in the cytoskeleton and spindle, is significantly enriched in GTPase activity and protein binding, and regulates cell division and mitosis in chromosome segregation and cell cycle progression.

Comprehensive analysis revealed that different tsRNA groups exhibit distinct functional biases in subcellular localization and molecular pathways. The Other-1 group is primarily involved in the precise regulation of nuclear transcription and replication; the Other-22 group focuses on protein synthesis and transcriptional regulation, highlighting its role in the homeostasis of cell proliferation; the Other-36 group concentrates on the dynamic regulation of the cytoskeleton during mitosis.

This functional differentiation provides important clues for understanding the interactions between genomic functional modules and lays the foundation for further exploration of specific genes’ core roles in cellular homeostasis, proliferation, and genetic information transmission. Such comparative and integrative analyses help to accurately delineate gene regulatory networks in complex biological systems, offering significant references for elucidating the multi-level functional characteristics at the gene level and potential molecular mechanisms.

### Construction and analysis of “tsRNA-Notch signaling pathway target” regulatory network based on functional enrichment analysis

**Figure 6:**
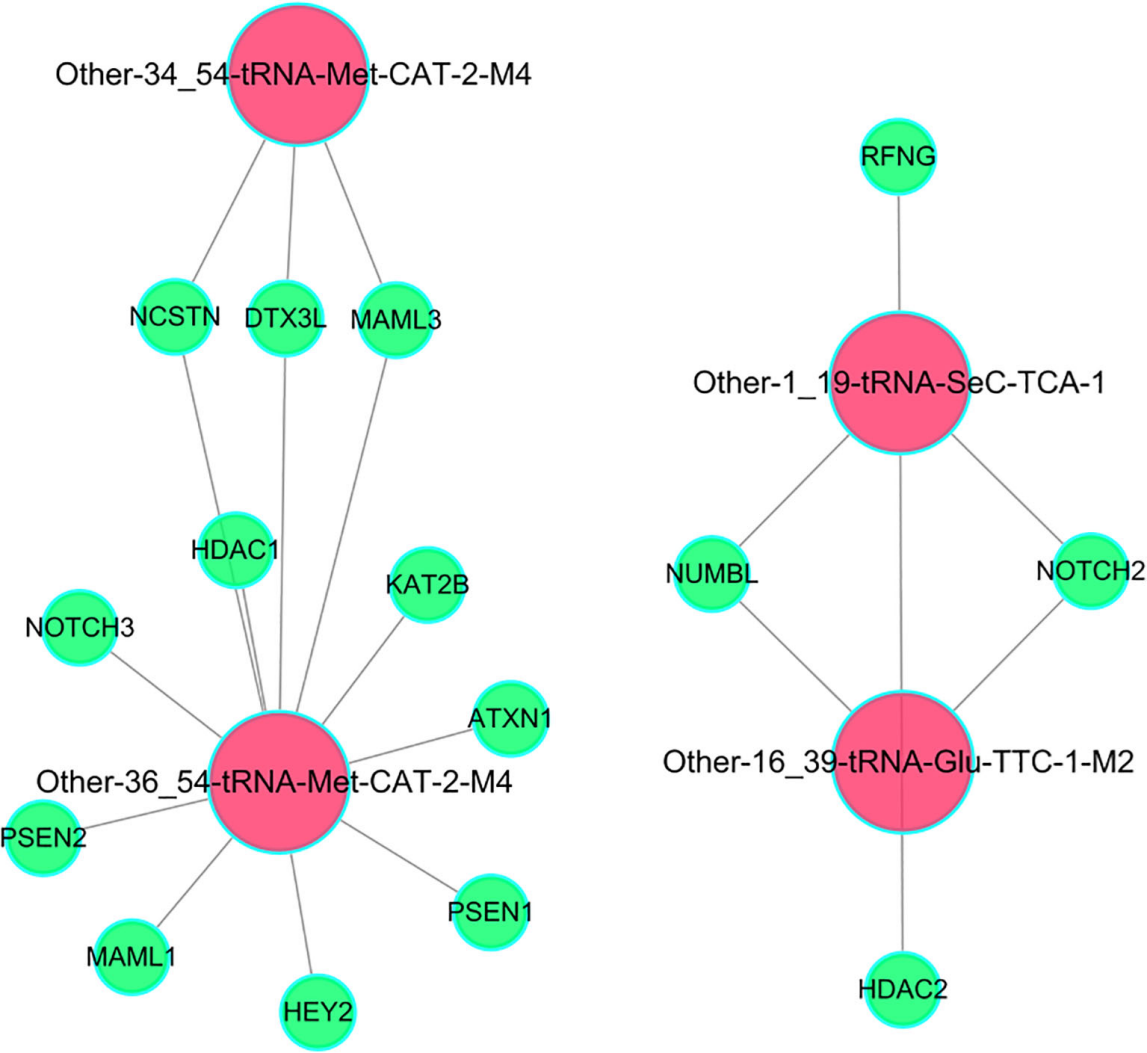
The regulatory network between differentially expressed tsRNAs and key target genes of Notch signaling pathway.

The gene regulatory network revealed that differentially expressed tsRNAs were enriched in key target genes of Notch signaling pathway, revealing the potential regulatory relationship between tsRNAs and Notch signaling pathway. A “tsRNA-Notch signaling pathway target” regulatory network was constructed based on the “total tsRNA-target gene” network using the target genes in the Notch signaling pathway identified through functional enrichment analysis. The results revealed that Other-34_54-tRNA-Met-CAT-2-M4, tRF-Met-CAT-2-M4, tRF-SeC-TCA-1 and Other-16_39-tRNA-Glu-TTC-1-M2 have regulatory interactions with key target genes of Notch signaling pathway, including ATXN1, HDAC1, RFNG, NUMBL, NOTCH3, HEY2, PSEN2, DTX3L, PSEN1, MAML1, MAML3, NCSTN, KAT2B, NOTCH2 and HDAC2 (**Figure 6**), suggesting these tsRNAs might regulate cell differentiation, proliferation and maturation. Besides, **Supplementary Figure S3** illustrated that tRF-Met-CAT-2-M4 and tRF-SeC-TCA-1 tsRNAs target distinct critical nodes within the Notch and TGF-β signaling pathways. In the Notch pathway, tRF-Met-CAT-2-M4 influences ADAM proteases, the PSE2/PSEN γ-secretase complex, NICD generation, and CSL-associated transcriptional coactivators (MAML/HATs) as well as corepressors (SMRT/HDAC). Conversely, tRF-SeC-TCA-1 modulates upstream regulators such as Dvl, Itch, and Numb (**Supplementary Figures S3A, B**). In the TGF-β pathway, tRF-Met-CAT-2-M4 regulates BMP/Activin receptors, Smad2/3/4 complexes, and downstream effectors including Id and p15 (**Supplementary Figure S3C**). Collectively, these results indicated that the two tsRNAs exert selective and cooperative regulation over key nodes in these signaling pathways.

### Clinical diagnostic value of key differentially expressed tsRNAs in MM patients and their association with renal injury

**Figure 7:**
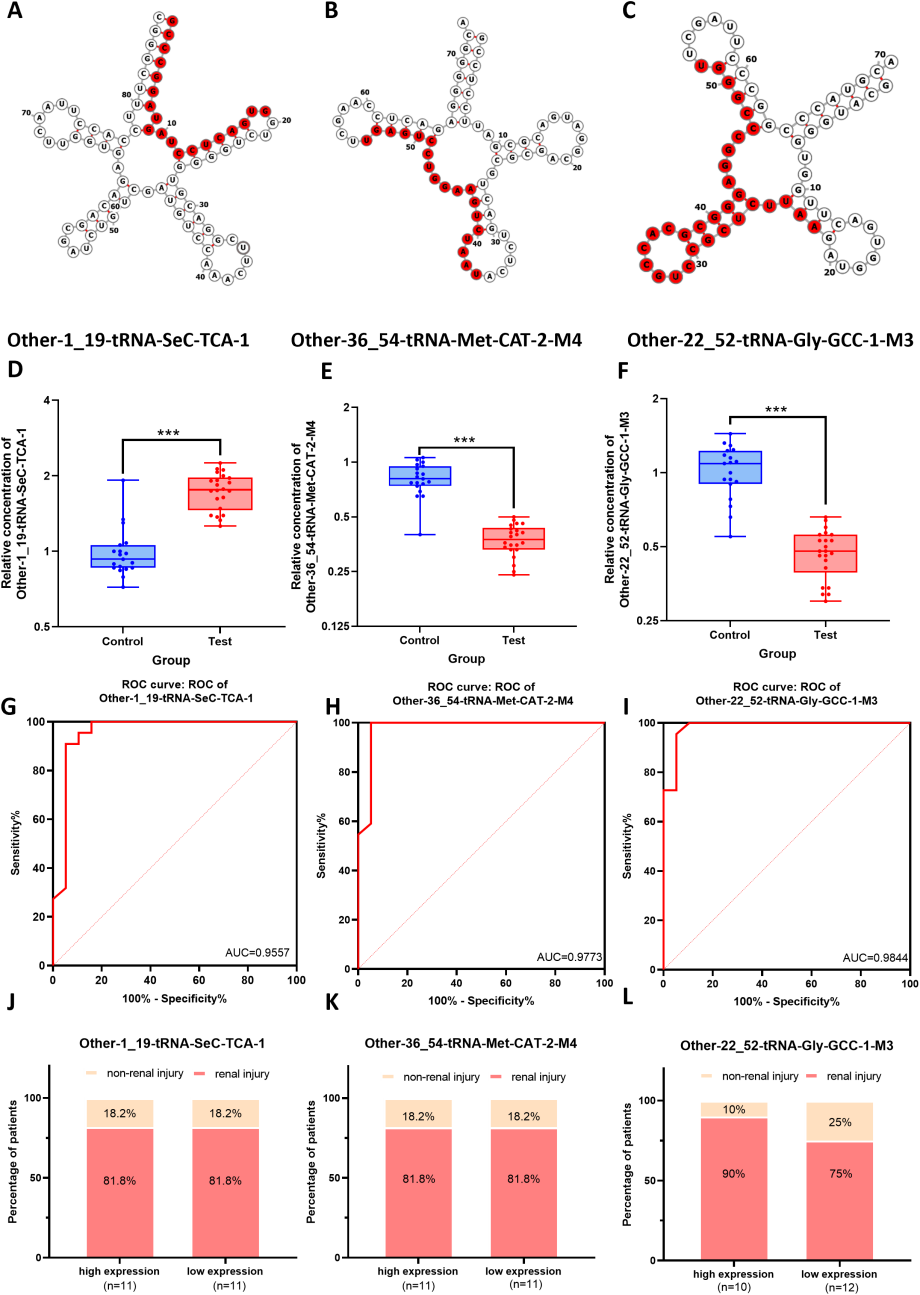
Clinical diagnostic value of the three key differentially expressed tsRNAs for renal injury of MM patients. **(A-C)** The structures of their corresponding tRNAs of tRF-SeC-TCA-1, tRF-Met-CAT-2-M4, and tRF-Gly-GCC-1-M3, with the tsRNA sequences highlighted in red. **(D-F)** The expression levels of tRF-SeC-TCA-1, tRF-Met-CAT-2-M4, and tRF-Gly-GCC-1-M3 in MM patients (Test) and health controls (Control). **(G-I)** The ROC curve analysis showing the AUC values of tRF-Gly-GCC-1-M3, tRF-Met-CAT-2-M4, and tRF-SeC-TCA-1 in the diagnosis of MM**. (J-L)** The associations of renal injury with the expression levels of tRNA-TCA1, tRNA-2M4, and tRNA-1M3 in MM.

To further validate the clinical relevance of the differentially expressed tsRNAs in MM, this study focused on the same three tsRNAs identified in the enrichment analysis:tRF-SeC-TCA-1,tRF-Gly-GCC-1-M3, tRF-Met-CAT-2-M4. The specific locations of these three tsRNAs in the secondary structures of their corresponding tRNAs were shown in **Figures 7A–C**. The secondary structures of the tRNAs were depicted using the classic cloverleaf model, clearly illustrating the stem-loop structures. The red markers precisely located each tRF within the parent tRNA molecule, reflecting the cleavage or generation of tRFs from specific regions of the parent tRNA, which may imply spatial specificity of their intracellular functions. The relevant data were sourced from the OncotRF database containing tRF information related to tumors. The expression levels of three tsRNAs in PBMCs from 22 patients with MM and 19 HCs were assessed using RT-qPCR. (see detailed baseline characteristics of the MM patients and healthy controls in **Supplementary Table S3**) The results indicated that the expression levels of tRF-Met-CAT-2-M4 and tRF-Gly-GCC-1-M3 were significantly downregulated in MM patients (p < 0.001), while the expression of tRF-SeC-TCA-1 was markedly upregulated (p < 0.001), consistent with the tsRNA sequencing results (**Figures 7D–F**). The ROC curve analysis showed that the areas under the curve (AUC) for tRF-Gly-GCC-1-M3, tRF-Met-CAT-2-M4, and tRF-SeC-TCA-1 in diagnosing multiple myeloma (MM) were 0.9844, 0.9773, and 0.9557, respectively, demonstrating that tRF-Gly-GCC-1-M3 exhibited the highest diagnostic efficacy (**Figures 7G–I**). Furthermore, the high and low expression groups of tRF-SeC-TCA-1 and tRF-Met-CAT-2-M4 exhibit no significant differences in the incidence of kidney injury, indicating that these two tRFs are not strongly associated with kidney damage. In contrast, the high expression group of tRF-Gly-GCC-1-M3 shows a notably lower rate of kidney injury, suggesting its potential as a biomarker for assessing kidney injury risk (**Figures 7J–L**).

### Correlation analysis of differentially expressed key tsRNAs with clinical markers in MM patients

**Figure 8:**
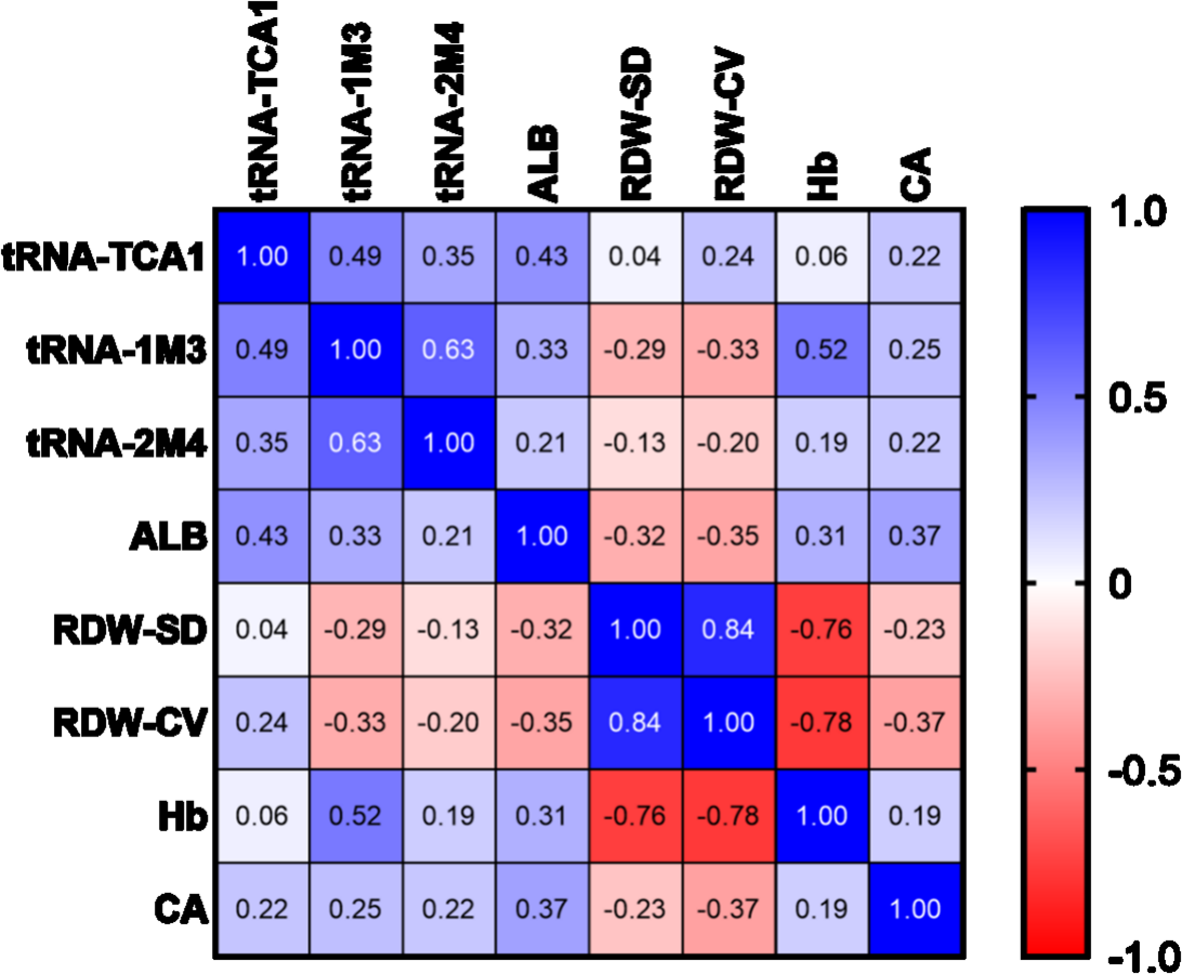
The positive correlations of three key differentially expressed tsRNAs with clinical markers in MM patients.

The associations between three key differentially expressed tsRNAs (tRF-Gly-GCC-1-M3, tRF-Met-CAT-2-M4, tRF-SeC-TCA-1) and clinical markers of MM were further explored through heatmap analysis. The findings revealed tRF-Gly-GCC-1-M3 showed a moderate positive correlation with hemoglobin (Hb) (*r* = 0.52), while tRF-SeC-TCA-1 exhibited a weak positive correlation with albumin (ALB) (*r* = 0.43), suggesting that these two tsRNAs may play roles in blood composition and nutritional metabolism. tRF-Gly-GCC-1-M3 and tRF-Met-CAT-2-M4 were negatively correlated with red blood cell distribution width (RDW-CV and RDW-SD), with tRF-Gly-GCC-1-M3 showing a more significant correlation with RDW-CV and RDW-SD (*r* = -0.33 and -0.29) (**Figure 8**).

## Discussion

In this study, we systematically profiled tsRNAs in PBMCs from patients with MM and healthy controls. Our data revealed a distinct tsRNA expression landscape in MM, with several tsRNAs showing marked dysregulation. These aberrantly expressed tsRNAs were associated with key signaling pathways, such as Notch, TGF-β, and PI3K-Akt; several exhibited strong diagnostic potential and significant correlations with clinical features. Together, these findings provide new insights into the potential roles of tsRNAs as biomarkers and regulatory molecules in MM, forming a basis for subsequent mechanistic exploration.

MM is a malignant hematologic tumor originating from plasma cells, characterized by typical clinical manifestations such as hypercalcemia, anemia, renal impairment, and osteolytic lesions, collectively referred to as CRAB symptoms (26, 27). Due to the lack of specific clinical symptoms and laboratory indicators, MM is often underdiagnosed in the early stages, and patients may seek medical attention in different departments due to nonspecific symptoms (28). Currently, early diagnosis and timely treatment can significantly improve the prognosis and extend remission periods, although MM is incurable (29). Therefore, the development of highly sensitive and specific non-invasive biomarkers is crucial for the early detection of MM.

As an emerging class of non-coding RNAs, tsRNAs have recently garnered widespread attention in gene expression regulation. Although dysregulation of tsRNAs has been observed in various cancers, autoimmune diseases, and metabolic disorders, the profiles and functions of tsRNAs in MM remain unexplored (30). This study analyzed tsRNA expression in PBMCs of MM patients and HCs using high-throughput sequencing, systematically revealing the expression profiles of tRFs & tiRNAs in MM patients and their potential diagnostic and predictive value. Our results showed that 148 tsRNAs were significantly upregulated and 63 were markedly downregulated in the PBMCs of MM patients, particularly subtypes such as tRF-5a, tRF-5b, and tRF-5c, suggesting that tsRNAs may play a critical regulatory role in the pathogenesis of MM. The high abundance of specific subtypes like tRF-5c in the 30–32 nt range indicated that they might influence the development of MM by participating in molecular mechanisms such as regulation of gene expression and cell cycle. ROC curve and heatmap analysis further validated the value of tRF-Gly-GCC-1-M3, tRF-Met-CAT-2-M4, and tRF-SeC-TCA-1 in predicting renal injury and serum marker levels of MM.

In disease diagnosis, MM patients often develop renal light-chain amyloidosis due to light-chain deposition, thus renal biopsy is considered the gold standard for diagnosis (26, 31). However, it is an invasive procedure with certain risks and high costs. Additionally, 24-hour urine protein quantification technique is widely used to measure light-chain protein in clinical practice, but it has several limitations, including timing inaccuracies, urine sample loss, and poor patient compliance (32). This study revealed that the incidence of renal injury reached 90% in the high-expression group of tRF-Gly-GCC-1-M3, significantly higher than the 75% in the low-expression group, suggesting its value of tRF-Gly-GCC-1-M3 as a potential biomarker for assessing renal injury risk in MM patients. This finding provides valuable insights for developing personalized treatment strategies and assists clinicians in evaluating patients’ renal function, potentially reducing the renal injury risk. Furthermore, although tRF-Met-CAT-2-M4 and tRF-SeC-TCA-1 showed no significant correlation with renal injury, their high AUC values (0.9773 and 0.9557, respectively) still indicated strong potential discriminatory ability in MM diagnosis. Based on the expression patterns of these tsRNAs, a multi-marker diagnostic model can be constructed to improve the accuracy and reliability of MM diagnosis. To further evaluate the additive diagnostic potential of these tsRNAs, we performed a combined ROC analysis integrating tRF-Gly-GCC-1-M3, tRF-Met-CAT-2-M4, and tRF-SeC-TCA-1 to assess their overall discriminative capacity in multiple myeloma (MM) diagnosis. The combined ROC curve demonstrated an overall discriminative performance comparable to that of the best single biomarker, without a statistically significant improvement (**Supplementary Figure S6**). This phenomenon is relatively common in multi-biomarker studies and may be attributed to biological correlations or overlapping information among biomarkers, resulting in a limited “effective information gain.” Internal validation using bootstrap resampling (n = 1000) demonstrated stable discriminative ability with a concentrated distribution of AUC values (**Supplementary Figure S7**), indicating high robustness and the absence of apparent overfitting. Considering that clinical implementation emphasizes simplicity and generalizability, a single-biomarker strategy may offer greater practicality when comparable diagnostic performance is achieved. Overall, the combined ROC analysis exhibited robust internal performance, providing strong support for the potential application value of tsRNAs in MM diagnosis. Further validation in larger, prospective, and multicenter cohorts is warranted to confirm its robustness and potential incremental value.

This study investigated the potential functions of differentially expressed tsRNAs in MM and revealed their roles in regulating critical biological processes including cell proliferation, apoptosis, differentiation, and microenvironment interactions by influencing multiple target genes. Our analysis predicted 1,112 target genes of three upregulated tsRNAs, and 4,190 target genes of five downregulated tsRNAs, in a total of 5,009 target genes after deduplication. The extensive network of target genes suggested that tsRNAs might regulate gene expression through various post-transcriptional mechanisms, thereby playing a role in the pathogenesis of MM. Regulatory network analysis further revealed that highly central tsRNAs, particularly Other-1_19-tRNA-SeC-TCA-1 and Other-13_30-tRNA-Lys-CTT-1-M2, functions as hubs regulating gene expression and modulating multiple signaling pathways, highlighting their critical role in the molecular mechanisms of MM. Our results provide potential biomarkers and therapeutic targets for molecular subtyping and personalized treatment of MM.

Accumulating evidence have also demonstrated that tsRNAs participate in cancer pathogenesis through regulation of key genes and signaling pathways. Huang et al. showed that tRF/miR-1280 inhibited JAG2 expression through direct targeting of its 3’UTR, consequently attenuating Notch signaling and inhibiting colorectal cancer cell proliferation (33). Zhu et al. reported tRF-5026a as a gastric cancer suppressor that inhibited cell proliferation via negative regulation of PTEN/PI3K/AKT signaling pathway (34). Xu et al. further confirmed that a miRNA-like t016 suppressed hyperactivation of MAPK signaling through downregulating the expression of *CACNA1d* gene, thereby inhibiting gastric cancer cell proliferation (35). These studies indicate that tsRNAs play a significant role in tumorigenesis and development through multiple signaling transduction pathways.

In this study, bioinformatics analysis revealed the potential role of tsRNAs in MM, particularly their regulatory impact on the Notch signaling pathway. KEGG pathway enrichment analysis indicated that differentially expressed tsRNAs may influence MM cell proliferation, survival, and metastasis by modulating signaling pathways such as TGF-β, Notch, MAPK, and PI3K-Akt. The critical role of the Notch signaling pathway in MM cell proliferation and bone marrow microenvironment remodeling provides a foundation for investigating its pathogenic mechanisms and identifying novel diagnostic and therapeutic targets.

To validate this hypothesis, we selected five representative genes from the Notch signaling pathway (HEY2, PSEN1, MAML1, NOTCH2, and NCSTN) and performed qRT-PCR analysis using the same peripheral blood mononuclear cell (PBMC) samples. Although we observed consistent trends in the expression levels of these genes between multiple myeloma patients and healthy controls, the differences did not reach statistical significance (p > 0.05), likely due to the limited sample size (10 patients and 10 controls), as shown in **Supplementary Figure S4**. Nevertheless, these preliminary data provide valuable insights and lend experimental support to our bioinformatics prediction that dysregulated tsRNAs may be involved in modulating the Notch signaling pathway in multiple myeloma. Future studies should not only expand the clinical cohort to confirm the reproducibility and robustness of these findings but also perform gain- and loss-of-function experiments in multiple myeloma–related cell lines and animal models. Such experiments could systematically evaluate how key tsRNAs influence critical components of the Notch signaling cascade, including HES/HEY transcriptional activity, γ-secretase complex function, and NICD expression levels. Furthermore, integrating multi-omics approaches—such as transcriptomic, proteomic, and tsRNA-interactome analyses—may help elucidate the mechanistic axis through which tsRNAs regulate Notch signaling, thereby contributing to tumor progression and immune microenvironment remodeling.

Despite the lack of statistical significance in the current results, these preliminary data provide valuable insights into the potential relationship between tsRNAs and the Notch signaling pathway and offer important directions for future research.

This study provides valuable preliminary insights but also has several limitations. The sample size was relatively small, and all participants were recruited from a single center, which may limit the statistical power and generalizability of the findings. Therefore, further validation in larger, multicenter cohorts that include patients across different disease stages and molecular subtypes is required to ensure robustness and reproducibility. Although high-throughput sequencing combined with bioinformatic analyses enabled a systematic characterization of tsRNA expression profiles in MM, functional studies are still needed to elucidate the precise biological roles and molecular mechanisms of key tsRNAs. Moreover, the detection of tsRNAs has not yet been fully standardized, which could introduce technical variability and affect data reproducibility. Future research should thus expand cohort size, incorporate comprehensive functional validation, and optimize detection and standardization procedures to strengthen the reliability and translational potential of tsRNAs as biomarkers.

Building upon these findings, our study provides a foundation for further functional exploration. In future work, we plan to investigate the biological roles of representative tsRNAs identified in this study, such as Other-1_19-tRNA-SeC-TCA-1 and Other-13_30-tRNA-Lys-CTT-1-M2, using multiple myeloma (MM) cell lines. Gain- and loss-of-function experiments will be conducted to assess their effects on MM cell proliferation, apoptosis, and migration. Furthermore, downstream regulatory mechanisms will be explored by examining canonical Notch and TGF-β signaling activities through qPCR, Western blot, and dual-luciferase reporter assays. These studies are expected to provide essential *in vitro* and *in vivo* evidence to validate the predicted regulatory functions of tsRNAs, thereby enhancing the biological relevance of our findings and supporting their potential clinical translation in MM.

In conclusion, this study revealed aberrant tsRNA expression profiles in MM and identified Other-22_52-tRNA-Gly-GCC-1-M3, Other-36_54-tRNA-Met-CAT-2-M4, and Other-1_19-tRNA-SeC-TCA-1 as promising noninvasive diagnostic biomarkers, with Other-22_52-tRNA-Gly-GCC-1-M3 showing particular value for renal injury assessment. These tsRNAs may act as novel regulatory molecules influencing multiple signaling cascades through multi-gene interactions, potentially contributing to MM pathogenesis and progression. Collectively, our findings provide new perspectives for tsRNA-based early diagnosis, treatment monitoring, and personalized therapy in MM, while establishing a foundation for their further development as tumor biomarkers.

## Data Availability

The data involved in this study are publicly available. This data can be found in the Gene Expression Omnibus (GEO), accession number: GSE311837.
