# Supplementary material for: Peripheral blood tRNA-derived fragments as novel noninvasive biomarkers for diagnosis and prognostic stratification in multiple myeloma
Source: Front Immunol. 2025 Dec 3;16:1650510. doi: 10.3389/fimmu.2025.1650510 (PMC12709168; doi:10.3389/fimmu.2025.1650510)
Supplement: Supplementary file 1 [file Table1.docx]

**Supplementary Materials for Peripheral Blood tRNA-derived Fragments as Novel Noninvasive Biomarkers for Diagnosis and Prognostic Stratification in Multiple Myeloma**

**Table S1. Primer sequences for RT-qPCR.**

| Gene | Primer sequences (5’→3’) | Annealing temperature (℃) | Product length (bp) |
| --- | --- | --- | --- |
| U6 | Former: GCTTCGGCAGCACATATACTAAAAT | 60 | 89 |
|  | Reverse: CGCTTCACGAATTTGCGTGTCAT |  |  |
| 1:19-tRNA-SeC-TCA-1 | Former: TTCTACAGTCCGACGATCGC | 60 | 46 |
|  | Reverse: TTCCGATCTCACTGAGGATCAT |  |  |
| 22:52-tRNA-Gly-GCC-1-M3 | Former: GACGATCAATTCTCGCCTGC | 60 | 54 |
|  | Reverse: TGTGCTCTTCCGATCTACCCG |  |  |
| 36:54-tRNA-Met-CAT-2-M4 | Former: TCCGACGATCTAATCTGAAGGT | 60 | 48 |
|  | Reverse: ACGTGTGCTCTTCCGATCTACT |  |  |


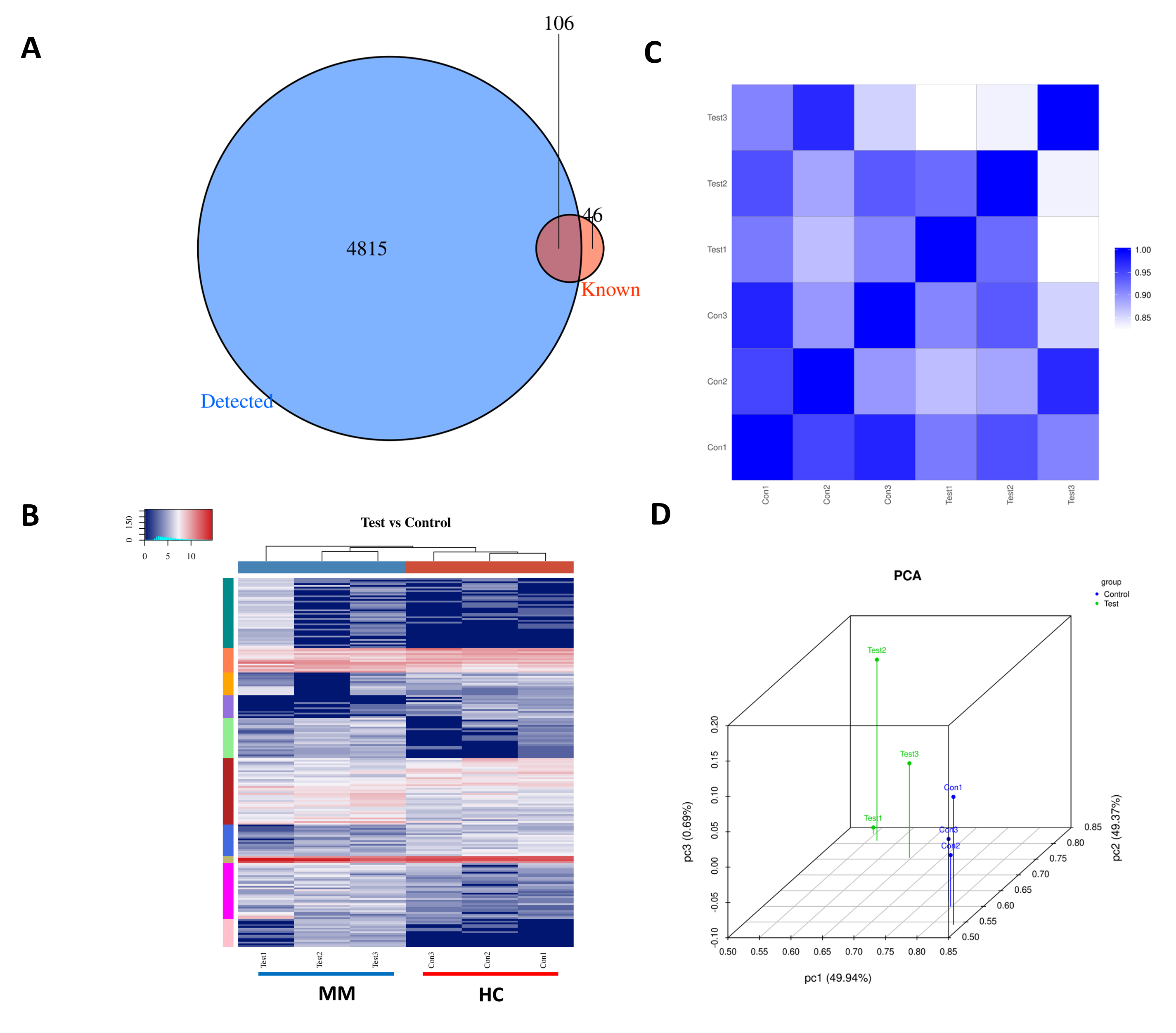


**Figure S1.** (A) Venn diagrams of known and unknown tsRNAs. (B) Heatmap of total differentially expressed tsRNAs between groups. (C) Correlation heatmap between samples. (D) Principal component analysis of tsNRA in two groups of samples.

**Table S2.** Sequencing information of 8 candidate tsRNAs.

| **tRF** **ID** | **tRF** **Sequence** | **tRF** **Length** | **Type** | **Fold** **Change** | **CPM** |
| --- | --- | --- | --- | --- | --- |
| Other-16-33-tRNA-iMet-CAT-1-M2 | CGGAAGCGTGCTGGGCCC | 18 | Other | 3.14 | 186.26 |
| Other-13-30-tRNA-Lys-CTT-1-M2 | CAGTCGGTAGAGCATGGG | 18 | Other | 2.24 | 219.99 |
| Other-1-19-tRNA-SeC-TCA-1 | GCCCGGATGATCCTCAGTG | 19 | Other | 3.53 | 1205.13 |
| Other-22-52-tRNA-Gly-GCC-1-M3 | AATTCTCGCCTGCCACGCGGGAGGCCCGGGT | 31 | Other | 0.39 | 73.57 |
| Other-36-54-tRNA-Met-CAT-2-M4 | TAATCTGAAGGTCCTGAGT | 19 | Other | 0.45 | 674.67 |
| Other-16-39-tRNA-Glu-TTC-1-M2 | CGGTTAGGATTCCTGGTTTTCACC | 24 | Other | 0.46 | 272.57 |
| Other-34-54-tRNA-Met-CAT-2-M4 | CATAATCTGAAGGTCCTGAGT | 21 | Other | 0.46 | 626.35 |
| Other-17-42-tRNA-Glu-CTC-1-M2 | GGTTAGGATTCGGCGCTCTCACCGCC | 26 | Other | 0.48 | 326.23 |

Abbreviation: tsRNA, transfer RNA-derived small noncoding RNA; tRF, transfer RNA-derived fragment.


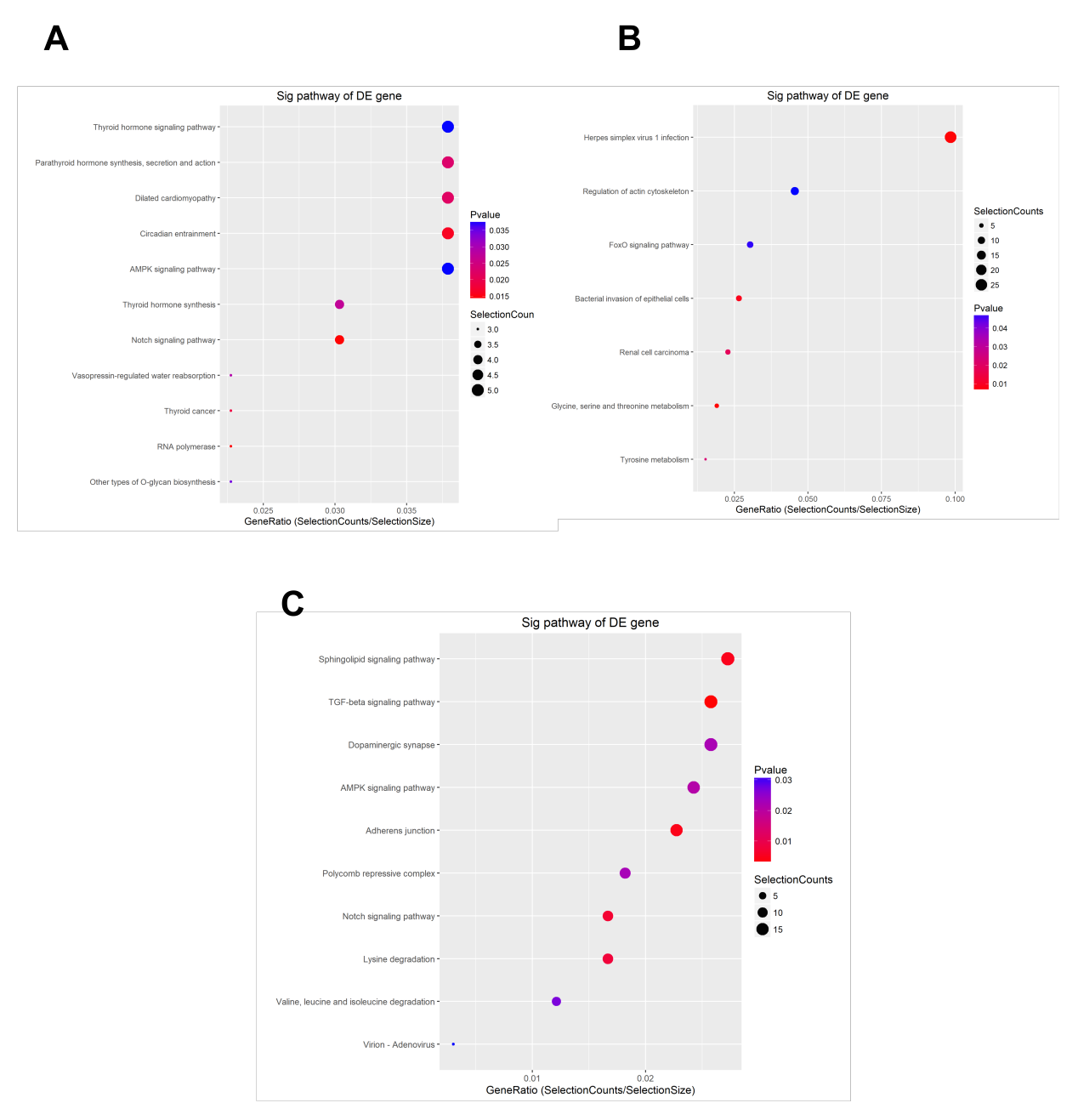


**Figure S2.** Functional enrichment analysis from KEGG data for target genes of three differentially expressed key tsRNAs of MM patients, including **(A)** Other-1_19-tRNA-SeC-TCA-1, **(B)** Other-22_52-tRNA-Gly-GCC-1-M3 and **(C)** Other-36_54-tRNA-Met-CAT-2-M4.


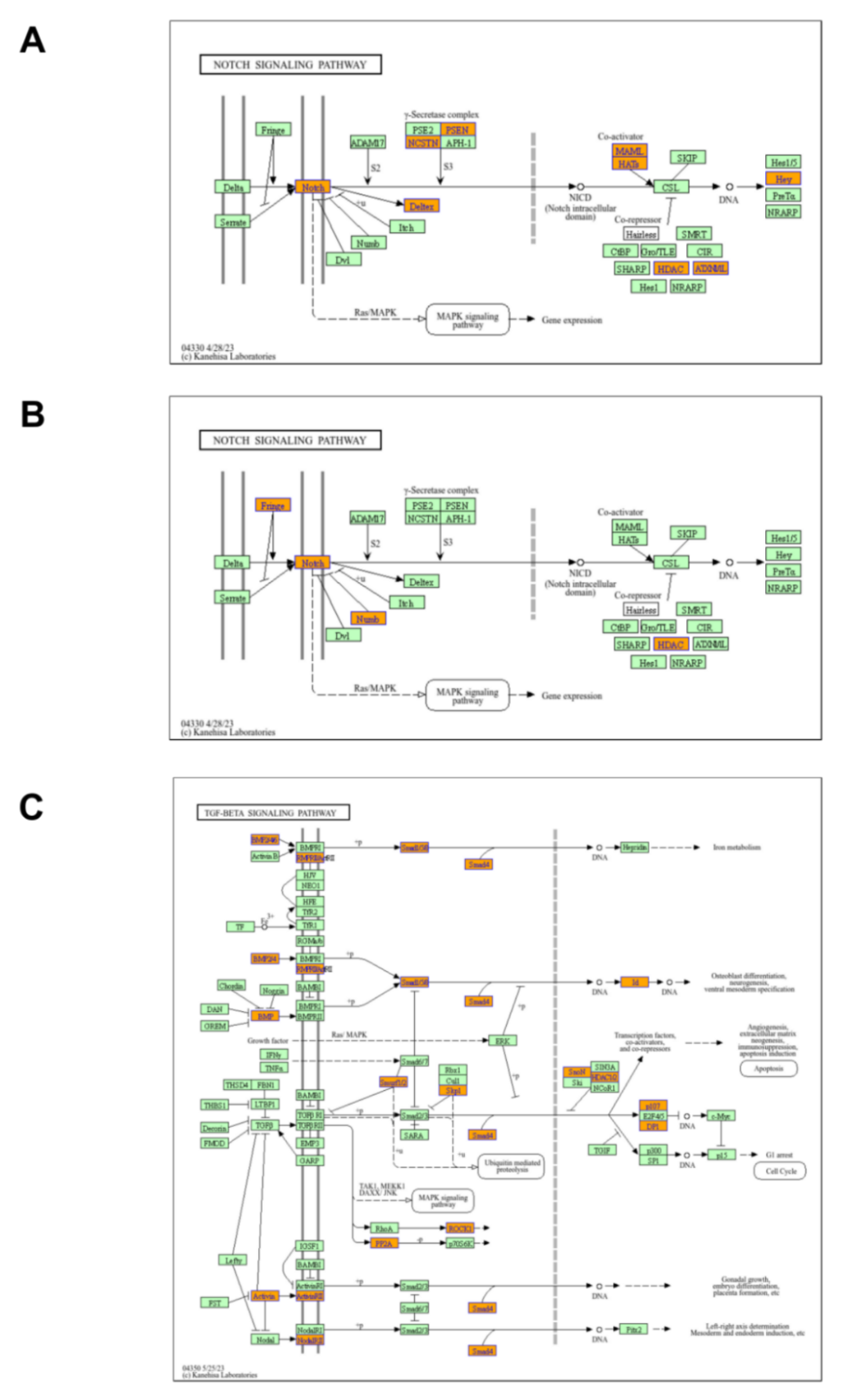


**Figure S3.** Modulation of Notch and TGF-β signaling pathways by Other-1_19-tRNA-SeC-TCA-1 tRF and Other-36_54-tRNA-Met-CAT-2-M4 tRF. **(A-B)** Mapping of Notch signaling pathway. Red marked nodes are associated with **(A)** Other-36_54-tRNA-Met-CAT-2-M4 tRF, and **(B)** Other-1_19-tRNA-SeC-TCA-1 tRF. **(C)** Mapping of TGF-β signaling pathway. Red marked nodes are associated with Other-36_54-tRNA-Met-CAT-2-M4 tRF.

**Table S3.**Baseline table of clinical data for control group vs patient group

| Variables, M (Q₁, Q₃) | Total (n = 41) | Con (n = 19) | Test (n = 22) | Statistic | *P* |
| --- | --- | --- | --- | --- | --- |
|  |  |  |  |  |  |
| Year | 64.00 (57.00, 72.00) | 64.00 (59.50, 71.00) | 64.50 (56.25, 72.75) | Z=-0.20 | 0.844 |
| Hb(g/L) | 121.00 (91.00, 140.00) | 140.00 (130.50, 147.50) | 91.00 (56.50, 112.00) | Z=-4.85 | **<.001** |
| WBC（10^9^/L） | 5.40 (4.56, 7.32) | 5.77 (4.96, 7.19) | 5.10 (3.75, 7.83) | Z=-0.61 | 0.543 |
| Neutrophil（10^9^/L） | 3.40 (2.61, 4.72) | 3.42 (2.86, 4.43) | 3.31 (2.43, 5.65) | Z=-0.16 | 0.875 |
| Lymphocyte（10^9^/L） | 1.51 (1.04, 1.80) | 1.78 (1.58, 2.13) | 1.11 (0.85, 1.48) | Z=-3.96 | **<.001** |
| PLT（10^9^/L） | 193.00 (162.00, 218.00) | 195.00 (179.00, 233.50) | 175.00 (121.50, 215.75) | Z=-1.33 | 0.182 |
| TP(g/L) | 70.90 (66.40, 74.70) | 70.90 (68.70, 73.60) | 70.50 (62.32, 96.48) | Z=-0.09 | 0.927 |
| ALB(g/L) | 42.10 (33.00, 45.60) | 45.60 (44.10, 47.10) | 33.15 (30.78, 38.15) | Z=-4.54 | **<.001** |
| ALT(U/L) | 20.00 (14.00, 27.00) | 21.00 (15.50, 26.50) | 18.50 (11.25, 34.25) | Z=-0.97 | 0.333 |
| AST(U/L) | 22.00 (18.00, 27.00) | 22.00 (18.50, 26.50) | 22.00 (17.00, 27.50) | Z=-0.07 | 0.948 |
| Crea(µmol/L) | 74.00 (61.00, 138.00) | 60.00 (52.00, 68.50) | 130.00 (75.00, 217.25) | Z=-4.16 | **<.001** |
| BUN (mmol/L) | 6.79 (5.27, 8.42) | 6.10 (4.90, 7.55) | 7.35 (5.97, 12.75) | Z=-1.84 | 0.065 |
| UA (µmol/L) | 351.00 (270.00, 453.00) | 306.00 (263.50, 350.00) | 434.50 (352.50, 591.75) | Z=-2.95 | **0.003** |
| BUN/UA | 0.02 (0.01, 0.03) | 0.02 (0.02, 0.02) | 0.02 (0.01, 0.03) | Z=-0.40 | 0.688 |
| Gender, n(%) |  |  |  | χ²=1.77 | 0.183 |
| 1 | 26 (63.41) | 10 (52.63) | 16 (72.73) |  |  |
| 2 | 15 (36.59) | 9 (47.37) | 6 (27.27) |  |  |
| BMG (mg/L) |  |  | 11.33 (5.00, 20.64) |  |  |
| eGFR（mL·min⁻¹·1.73 m⁻²） |  |  | 49.85 (15.77, 77.92) |  |  |
| 24hUTP（g/24 h） |  |  | 0.90 (0.27, 3.82) |  |  |
| uFLC κ/λ |  |  | 3.19 (0.03, 15.22) |  |  |
| UPro/FLC |  |  | 0.05 (0.01, 36.46) |  |  |
| Z: Mann-Whitney test, χ²: Chi-square test | | | | | |
| M: Median, Q₁: 1st Quartile, Q₃: 3st Quartile | | | | | |
|  | | | | | |


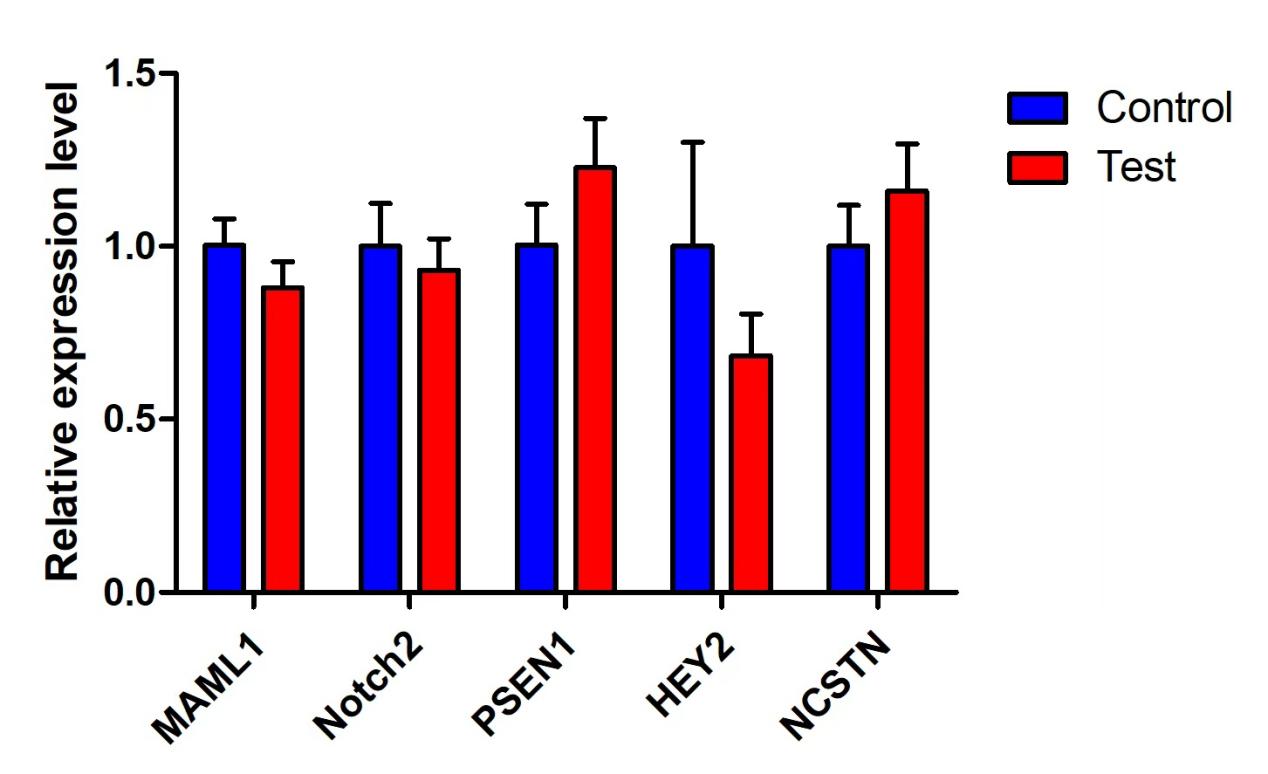


**Figure S4.** Relative expression levels of Notch signaling pathway-related genes (MAML1, Notch2, PSEN1, HEY2, NCSTN) in PBMCs. Data were obtained by qRT-PCR and compared between the control and test groups. Blue bars represent the control group, and red bars represent the test group. Data are presented as the mean ± standard deviation. No statistically significant differences were observed.


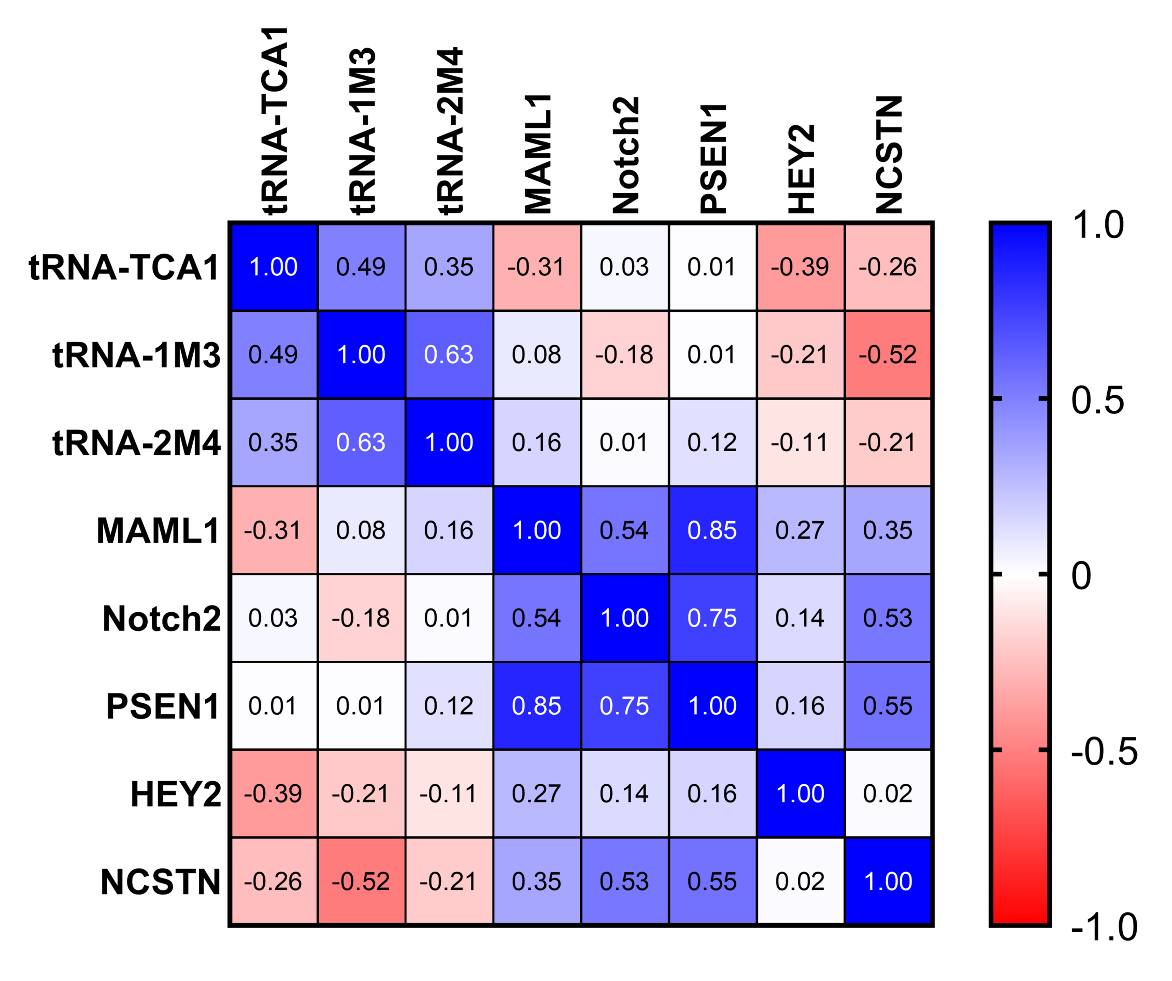


**Figure S5.**Correlation heatmap between tsRNA isoacceptors and Notch pathway–related genes. Spearman’s correlation coefficients (ρ) were computed between three tsRNA isoacceptors (tRNA-TCA1, tRNA-1M3, and tRNA-2M4) and five Notch pathway genes (MAML1, Notch2, PSEN1, HEY2, and NCSTN). The color scale ranges from −1 (red) to 1 (blue). tsRNA isoacceptors displayed moderate positive correlations (ρ = 0.35–0.63), whereas strong correlations were found among Notch genes, especially MAML1–PSEN1 (ρ = 0.85) and Notch2–PSEN1 (ρ = 0.75). A moderate negative correlation was observed between tRNA-1M3 and NCSTN (ρ = −0.52).

**
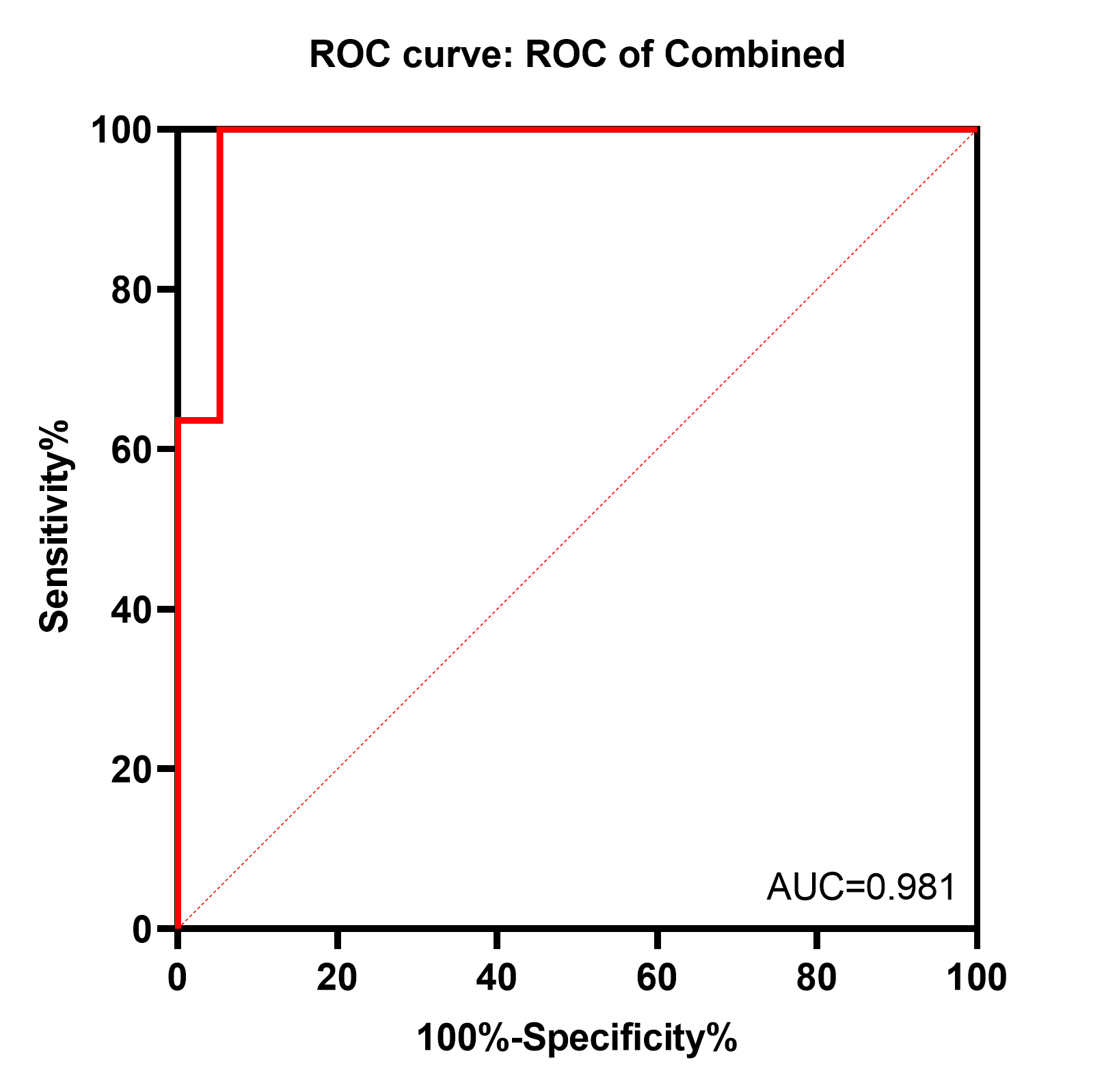
**

**Figure S6 .** **Receiver operating characteristic (ROC) curve of the combined model incorporating tRF-Gly-GCC-1-M3, tRF-Met-CAT-2-M4, and tRF-SeC-TCA-1 for the diagnosis of multiple myeloma (MM).**The ROC curve was derived from a logistic regression model constructed using the three significantly dysregulated tsRNAs. The area under the curve (AUC) represents the overall discriminatory performance of the combined model in distinguishing MM patients from healthy controls.


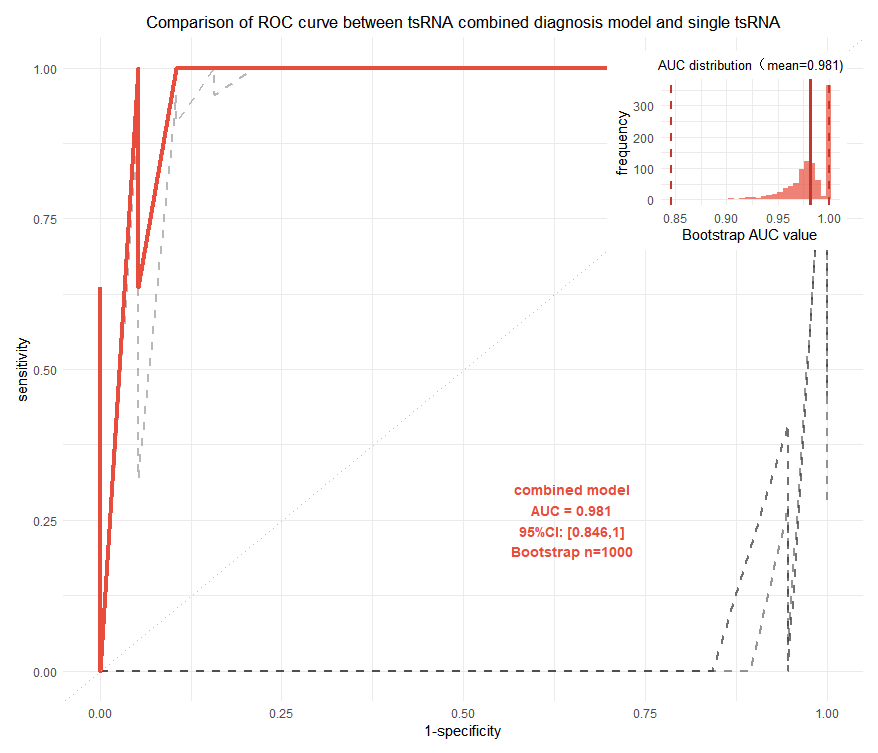


**Figure S7. ROC curve comparison between the tsRNA combined diagnostic model and the single tsRNA model.**

The red solid line represents the combined model and the gray dashed line indicates the single tsRNA. The combined model achieved an AUC of 0.981 (95% CI: 0.846–1.000), validated by bootstrap resampling (n=1000). The inset shows the bootstrap AUC distribution (mean = 0.981), indicating high model stability without evidence of overfitting.
